# Clavicular Head Subluxation Resulting in Tracheal Compromise in an Osteogenesis Imperfecta Type III Patient: A Case Report

**DOI:** 10.7759/cureus.95006

**Published:** 2025-10-20

**Authors:** Abraham Oommen, Dillon Froass, Mark Dobish

**Affiliations:** 1 Anesthesiology and Perioperative Medicine, Nemours Children's Health System, Wilmington, USA; 2 Anesthesiology and Perioperative Medicine, Ohio State University Wexner Medical Center, Columbus, USA; 3 Anesthesiology, MedStar Georgetown University Hospital, Washington D.C., USA

**Keywords:** critical airway, osteogenesis imperfecta, skeletal dysplasia, subluxed sternoclavicular joint, tracheal compression

## Abstract

Osteogenesis imperfecta (OI) poses many known challenges to anesthesiologists; however, structural and functional abnormalities of the trachea have not previously been described. OI results in fragile and brittle bones due to a defect in type 1 collagen production. Perioperative anesthetic considerations include risk of fracture with patient positioning, potential for difficult airway, and managing restrictive lung disease in conjunction with spinal deformities. Our case describes an adolescent male patient with OI type III who was found to have significant tracheal compromise and airway obstruction with the induction of general anesthesia for spinal fusion. Bronchoscopy performed after intubation revealed a compression and tortuosity of the distal trachea, confirmed with a postoperative computed tomography angiogram. Subluxation of the right clavicular head caused tracheal compression and narrowing. This is the first reported case of a patient with OI type III with undiagnosed tracheal compression and deviation secondary to clavicular head subluxation.

## Introduction

Osteogenesis imperfecta (OI) is an autosomal dominant inherited disorder affecting bone growth due to abnormal type 1 collagen, occurring in one in 15,000 to 20,000 births. OI is one of the most common skeletal dysplasias affecting 6-7 individuals per 100,000 worldwide [1]. There are currently eight known subtypes of OI. Type I occurs most frequently and is the most benign form [2]. Type III is characterized by persistent fractures, growth delay, and kyphoscoliosis leading to cardiac and respiratory comorbidities. These presentations pose significant challenges to anesthesiologists, especially the increased risk of pathologic fractures with positioning. Cardiac anomalies and increased bleeding are also described in the medical literature; however, tracheal abnormalities have never been reported [3]. The life expectancy of patients with OI is increasing with improved access to healthcare, and anesthesia providers should be familiar with the common comorbidities. We report an interesting case of a patient with OI type III having a critical airway event because of significant tracheal narrowing at the thoracic inlet secondary to a subluxed sternoclavicular joint. This report aims to raise suspicion of distal tracheobronchial pathology in OI patients when ventilation remains difficult despite successful intubation. Written authorization under the Health Insurance Portability and Accountability Act was obtained from the patient to publish this case report.

## Case presentation

A 19-year-old male patient with OI type III, restrictive lung disease, and thoracolumbar scoliosis presented to our institution for a T2-L4 posterior spinal fusion (PSF). His past surgical history includes 18 surgical procedures, mostly orthopedic in nature for long bone fractures. The single anesthetic at our institution consisted of the application of a halo traction device performed 20 days prior to the PSF. Application of the halo traction device was performed under general anesthesia with a supraglottic airway (SGA). Induction was achieved with intravenous propofol and maintenance with sevoflurane in a 50:50 air-to-oxygen mixture. The patient's anesthetic history revealed no prior known history of difficult intubation, including an uneventful intubation five years ago at an outside facility using a 5.5 mm cuffed endotracheal tube (ETT).

**Figure 1:**
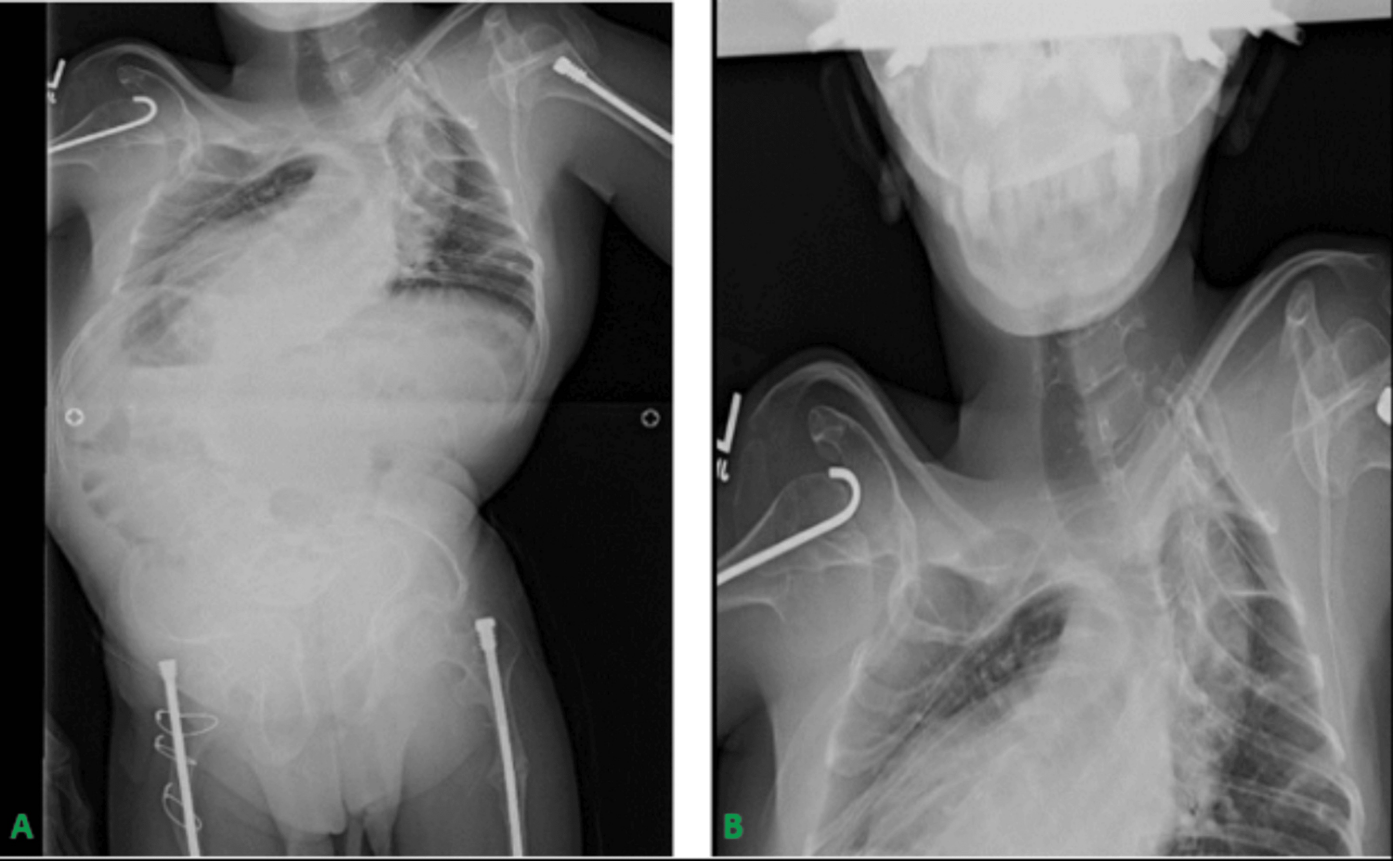
(A) Plain radiograph revealing a crowded thoracic cavity with severe kyphoscoliosis, rendering interpretation of any airway abnormalities difficult. (B) Enhanced view of plain radiograph focusing on the chest.

Preoperative cervical MRI revealed severe cervical kyphosis, basilar invagination, severe dextro-curvature of the thoracic spine from T1 to T12 (Cobb angle 98°), and a levo-curvature of the lumbar spine from T12 to L5 (Cobb angle 83°). A thoracic kyphosis was observed, but difficult to measure due to the presence of severe scoliosis. Echocardiography was normal. The trachea was not well visualized in any of the preoperative plain radiographs due to overcrowding of intrathoracic structures (Figures 1A, 1B). On physical examination, we met an alert young man with pectus carinatum and limited neck movement with a height of 3'6'' and a weight of 25.8 kg.

**Figure 2:**
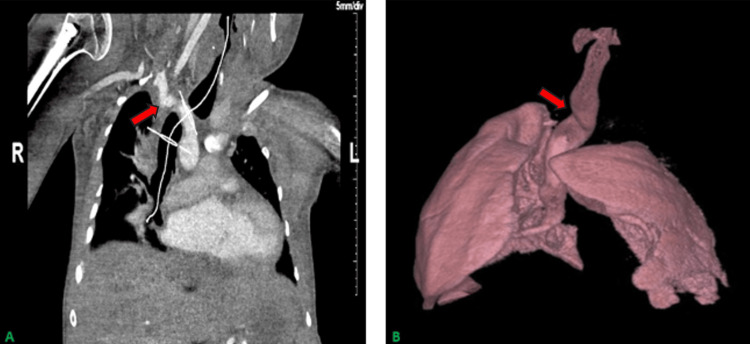
(A) Orthogonal CT image showing tracheal impingement by the posteriorly subluxed clavicular head. (B) Lung and tracheal reconstruction showing evidence of tracheal buckling. CT: computed tomography

The patient was preoxygenated, and general anesthesia was induced with a bolus dose of propofol and inhaled sevoflurane. Shortly after loss of consciousness, bag mask ventilation became difficult, and an oral airway was placed. Chest rise improved, but a capnogram showing effective ventilation was not reestablished. Direct laryngoscopy was performed using a Macintosh #3 blade, and a 6.5 mm cuffed ETT was passed through the vocal cords easily. Advancement of the ETT further into the trachea proved difficult. At this time, a capnogram was appreciated, but it showed a marked obstructive waveform. Oxygen saturations reached a nadir of 82% and remained below 90% for approximately five minutes as the airway was secured. Evaluation of the trachea with flexible bronchoscopy (FB) was performed given difficulty with ETT advancement. A rigid bronchoscope was considered, but the hyperextension required for rigid bronchoscopy would almost certainly result in a cervical fracture in our patient. FB revealed a deviated, torturous, and narrow trachea. Using the FB as a guidewire, the ETT was gently railroaded past the diseased tracheal segment, and the tip of the ETT was positioned proximal to the carina. This maneuver resulted in the return of normal saturations. A portable chest X-ray was taken to confirm the final position of the ETT. The PSF lasted for eight hours and remained uneventful, and the trachea was extubated at the conclusion of the case. Postoperatively, a computed tomography angiogram (CTA) of the chest was obtained to further evaluate the tracheal abnormality. The CTA revealed that at its narrowest point, the trachea was compressed to 7 mm in diameter (Figures 2A, 2B) by posterior subluxation of the right clavicular head with posterior displacement of the distal innominate artery.

## Discussion

Patients with OI face various perioperative and procedural challenges, including difficult intravenous access, issues with measuring blood pressure, and complications with airway management. In the management of patients with mucopolysaccharidoses presenting with similar manifestations to OI, forward tongue retraction using sterile gauze, a ringed forceps, or a suture directly through the tongue has been reported before placement of an oral airway to prevent the possibility of the posterior part of the tongue obstructing the airway [4,5].

The second notable feature of this case was the unexpected difficulty in advancing the ETT, given previous uneventful anesthetics. Patients with OI are also not known to have tracheal abnormalities. Although patients with OI often experience upper airway challenges related to limited neck mobility or a prominent sternum, kyphoscoliosis itself is not considered a risk factor for tracheal abnormalities. Described techniques to intubate the trachea in OI patients include placement of an intubating laryngeal mask airway (LMA), use of a video laryngoscope, or FB to minimize bony manipulation [6,7]. Our patient was in halo traction for 20 days before his PSF. There is a case report of difficulty with ventilation when using halo traction, which is a potential confounder [8]; however, the airway CTA provides evidence for an alternative explanation for lower airway obstruction.

Tracheal abnormalities, including deviation, buckling, and narrowing, are most well characterized in lysosomal storage disorders called mucopolysaccharidoses, most notably in Morquio syndrome (MS) [4,9,10]. In this condition, an enzyme deficiency leads to glycosaminoglycan accumulation, which disrupts bone and cartilage production, resulting in physical deformities such as gross skeletal dysplasia, progressive joint laxity, kyphoscoliosis, and spinal cord compression [11,12]. Although the exact pathogenesis of airway malformation in MS remains unknown, the prevailing theory implicates differential growth rates of the trachea, neck, and chest cavity, along with a narrow thoracic inlet and overcrowding of structures in the thoracic cavity, all of which contribute to marked airway distortion [10]. In MS patients, the innominate artery commonly traverses the trachea, which was also observed in our OI patient. Although tracheal abnormalities in MS patients are well recognized at our institution, the presence of similar obstructive tracheal pathology in this patient with OI was unanticipated.

**Figure 3:**
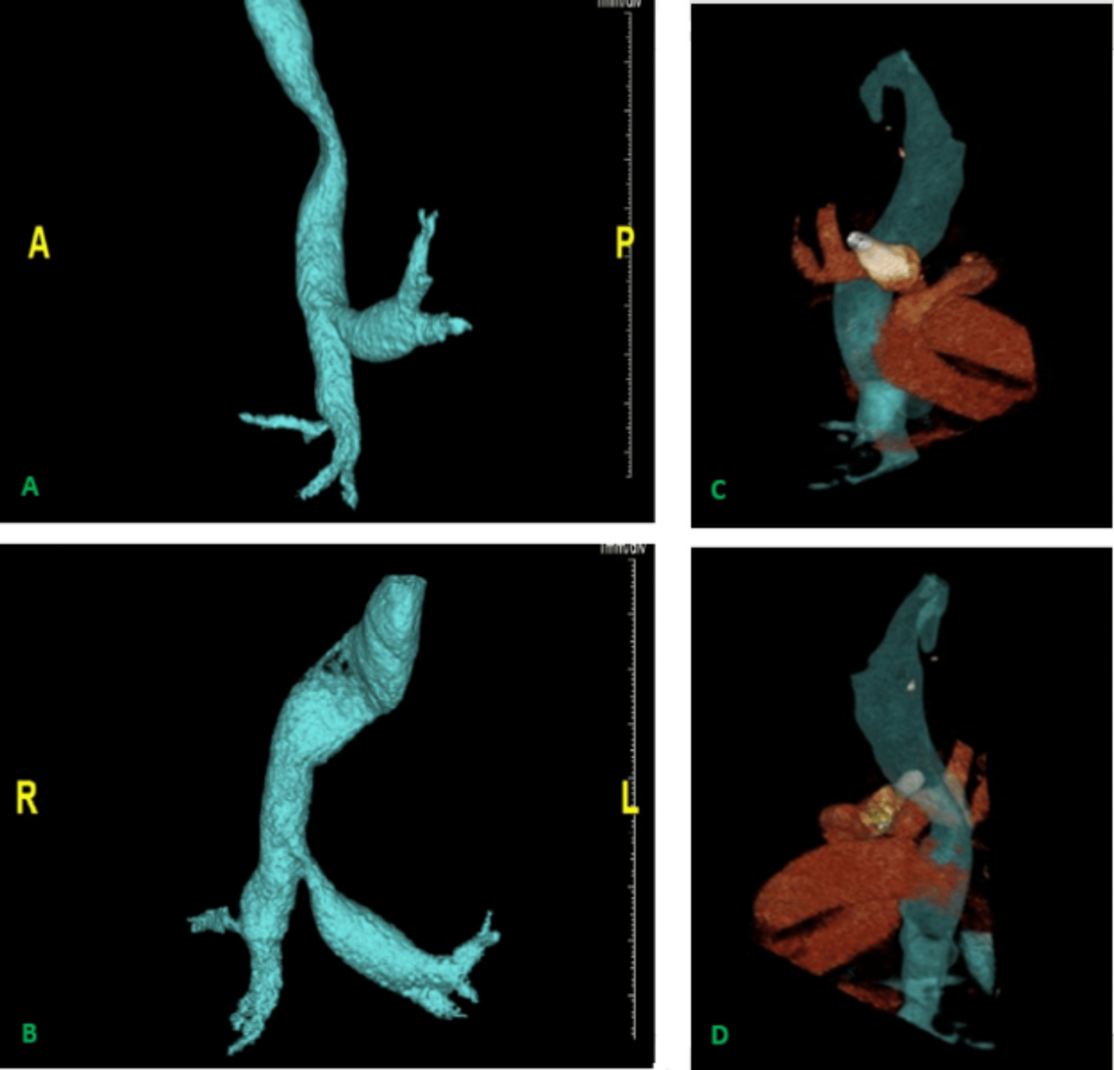
(A, B) Processed 3D image from CTA demonstrating tracheal pathology proximal to the carina. (C, D) Processed 3D image from CTA including the brachiocephalic artery and the clavicular head showing compression of the distal trachea. CTA: computed tomography angiogram

There was direct compression of the trachea by his subluxed right clavicular head, exacerbated by a posteriorly displaced innominate artery evident on CTA (Figures 3A-3D). Unlike patients with MS, no storage deposits are known to occur in OI. Nevertheless, there is still some overlap between the phenotypes of patients with these two diseases, namely, short stature, a narrow thoracic inlet, a small thoracic cavity, and a large tongue. Such features, combined with a kyphotic curve of the spine, are one explanation for the atypical anatomy seen in our patient. The distorted anatomy compromises the space available for airway and vessel development.

The tracheal abnormality observed in this patient may become increasingly prevalent as advances in medical therapy, particularly newer bisphosphonate treatments, allow patients with OI to live longer. As these patients are typically not very physically active, signs and symptoms of tracheal abnormalities may not be evident based on history alone. Advanced imaging modalities such as a CTA may be required to thoroughly evaluate the lower airway. Pulmonary function studies are not diagnostic for central airway obstruction but may be useful for follow-up of patients’ clinical symptoms and help dictate treatment [13].

In OI patients, any suspicious abnormality on chest radiographs or physical exam warrants additional evaluation for potential tracheal abnormality. OI patients often have limited exercise capability, making it difficult to assess cardiopulmonary reserve. Clinicians should have a low threshold for obtaining additional investigative studies. We recommend that patients with OI with significant overcrowding of the chest have a CTA with a focus on the large airways and major vessels. If a tracheal abnormality is noted on CTA, FB should be available in the room, and an otolaryngologist with advanced airway skills should be notified. Anesthesiologists should be prepared to manage airway challenges and recognize that successful intubation does not always resolve distal tracheobronchial obstruction.

## Conclusions

Patients with OI present several challenges during the perioperative period, one of which is the potential for airway issues. As OI patients age, conditions like thoracolumbar scoliosis, kyphoscoliosis, and other skeletal abnormalities-such as those observed in our case-can lead to compression of the distal trachea. Therefore, conducting a thorough preoperative assessment, including careful evaluation of chest radiographs, is essential for the safe management of these patients.
